# Association of habitual diet and bone marrow adipose tissue – magnetic resonance imaging in a population-based sample

**DOI:** 10.1186/s12937-026-01365-z

**Published:** 2026-07-08

**Authors:** Elena Grune, Dunja Hasic, Christopher L. Schlett, Fabian Bamberg, Annette Peters, Nina Wawro, Jakob Linseisen, Susanne Rospleszcz, Marc-Nicolas von Itter

**Affiliations:** 1 Department of Radiology, Medical Center and Faculty of Medicine, University of Freiburg, Freiburg, Germany; 2 Institute of Epidemiology, Helmholtz Zentrum München - German Research Center for Environmental Health (GmbH), Neuherberg, Germany; 3 Chair of Epidemiology, IBE, LMU Medizin, Ludwig-Maximilians-Universität München, Munich, Germany; 4 Department of Radiology and Nuclear Medicine, Luzerner Kantonsspital, Lucerne, Switzerland; 5 German Center for Diabetes Research (DZD), Neuherberg, Germany; 6 German Centre for Cardiovascular Research (DZHK e.V.), Partner Site Munich Heart Alliance, Munich, Germany; 7 Epidemiology, University of Augsburg, University Hospital Augsburg, Augsburg, Germany

**Keywords:** Bone marrow adipose tissue, Magnetic resonance imaging, Habitual diet, Population-based

## Abstract

**Background:**

Bone marrow adipose tissue (BMAT) is a metabolically active fat depot that may influence bone and hematopoietic function, yet its relationship with habitual diet remains largely unexplored at the population level. As diet represents an easily modifiable factor, a better understanding of its role in bone health and potential sex-specific differences is needed.

**Objective:**

We examined how habitual intake of energy-providing nutrients (fat, carbohydrates, protein, alcohol) and micronutrients (calcium, phosphorus, vitamin D) relates to imaging-derived measures of BMAT.

**Methods:**

In a sample from a population-based cohort, habitual diet was assessed based on a food frequency questionnaire and repeated 24 h recalls. Using magnetic resonance imaging (MRI) with a 2-point T1-weighted Dixon sequence, fat fraction of BMAT was quantified at vertebrae L1 and L2 in *N* = 297 participants (44% women, mean age 56.1 years, mean BMI 28.0 kg/m²) and at left and right femur in *N* = 163 participants (67% women, mean age 57.5 years, mean BMI 27.1 kg/m²) with available dietary data. Associations between habitual diet and BMAT were assessed using correlation analyses and linear regression models in the overall study population and stratified by sex, adjusting for age, BMI, physical activity, and diabetes status.

**Results:**

Mean vertebral and femoral BMAT were 54.8% and 86.5% in women, and 54.6% and 89.8% in men, respectively. Vertebral and femoral BMAT were positively correlated in women (ρ = 0.54, *p* < 0.001) and men (ρ = 0.36, *p* = 0.0096). Associations between habitual intake of energy-providing nutrients or micronutrients and BMAT were mostly non-significant across anatomical sites and sexes. Only in women, protein intake was positively associated with vertebral BMAT (β = 1.03, 95%CI [0.2, 1.9], *p* = 0.020, per percent of total energy intake). In men, an opposite effect direction was observed, which was not significant (β =-0.73, 95%CI [-1.6, 0.1], *p* = 0.096). For femoral BMAT, all associations were non-significant in either sex.

**Conclusion:**

In this population-based sample, habitual dietary intake of energy-providing nutrients and micronutrients showed no consistently significant association with vertebral or femoral BMAT in women and men. Opposite effect directions in men and women, as well as one significant association between protein intake and vertebral BMAT in women were observed in exploratory analyses. This may indicate potential site- and sex-specific differences, underlining the distinct biological properties of this adipose tissue depot.

**Supplementary Information:**

The online version contains supplementary material available at 10.1186/s12937-026-01365-z.

## Introduction

Bone marrow is a complex tissue that changes throughout life and in response to physiological stress or disease. It contains different cell types, including hematopoietic and stromal cells, which give rise to the various blood components and provide the structural framework of the marrow, respectively. The most abundant non-hematopoietic cells are bone marrow adipocytes, which form the bone marrow adipose tissue (BMAT) and modulate the development and differentiation of both hematopoietic and stromal cells [1].

Bone marrow adipocytes also interact with bone cells and actively participate in bone homeostasis by influencing the development of osteoblasts and osteoclasts [2]. Clinically, higher BMAT is consistently associated with lower bone mineral density and a higher risk of vertebral fractures [3]. This inverse relationship is particularly evident in postmenopausal osteoporosis, where estrogen deficiency drives both BMAT increase and accelerated bone resorption [4].

Beyond osteoporosis, BMAT has been more deeply investigated in conditions characterized by extreme nutritional states, including nutrient overload such as obesity and high-fat diet, but also caloric insufficiency as seen in anorexia nervosa [5, 6]. However, the relationship between dietary factors and BMAT, as well as the underlying mechanisms, remains poorly understood. Especially for carbohydrate intake, including glucose, evidence on BMAT remains limited, as most animal and clinical studies focused on bone homeostasis [7]. Similarly, the impact of dietary protein on bone health is unclear. Studies in animal models and humans exhibited very different settings and mostly focused on bone mineral density [8, 9].

At the level of micronutrients, calcium and phosphorus are essential for bone mineralization, while vitamin D is required for their absorption and utilization. Adequate calcium and vitamin D intake supports bone mineral density and reduces osteoporosis risk, whereas a high phosphorus intake relative to low calcium may increase parathyroid hormone levels and contribute to lower bone mineral density [10]. Direct effects of micronutrients on BMAT are poorly described, and most knowledge is based on indirect evidence from bone metabolism studies or animal models [7].

Overall, diet and nutrient status appear to be potentially important, yet complex, modulators of BMAT but are not well examined. Given that BMAT substantially varies by age, sex, and skeletal site [3], population-based studies with a balanced sex distribution, broad age range, and BMAT quantification across different regions are needed to better understand these interacting determinants. To facilitate comprehensive BMAT assessment across different skeletal sites, non-invasive imaging methods are required, with magnetic resonance imaging (MRI) providing a robust and well-established approach [11].

The KORA-MRI study offers such a framework, including participants who underwent both whole-body MRI and a comprehensive dietary assessment. Previous analyses in this subsample of a population-based cohort have established the correlation of BMAT with traditional cardiovascular risk factors [12], and have shown associations of habitual diet with skeletal muscle fat infiltration [13] and abdominal and hepatic adipose depots [14].

Building on this, while recognizing the specific properties of this adipose tissue depot, we aim to investigate overall and sex-specific associations of habitual dietary intake of energy-providing nutrients (fat, carbohydrates, protein, alcohol) and micronutrients (calcium, phosphorus, vitamin D) with MRI-based measures of BMAT.

## Methods

### Study sample

The analysis is based on cross-sectional data from the KORA-MRI study, a subsample of the KORA-FF4 study (“Cooperative Health Research in the Region of Augsburg”) conducted in 2013–2014. KORA-FF4 included 2279 participants as part of the second follow-up of the baseline KORA-S4 survey, in which originally 4261 participants aged 25–74 years were enrolled in 1999–2001. Details on design and sampling procedures of this population-based cohort have been described elsewhere [15].

The KORA-MRI study included 400 participants who underwent whole-body MRI as described previously [16]. Briefly, the study aimed to investigate systemic metabolic effects of glycemic impairment in middle-aged individuals. Participants with available data on glycemic status and consent to undergo MRI were eligible. Exclusion criteria were any contraindications to MRI, age over 73 years, and history of cardiovascular disease (myocardial infarction, stroke, or revascularization). No minimum age criterion was applied; however, due to the follow-up interval, participants were 39 years or older at the imaging visit. The MRI examination was conducted within three months of the initial visit at the study center, during which all participants completed a standardized face-to-face interview, physical examination, and blood sampling.

The study was approved by the ethics committee of Ludwig-Maximilians-University Munich (No. 498 − 12) and the Bavarian Chamber of Physicians (EC No. 06068). It was performed according to the Declaration of Helsinki, including written informed consent of all participants.

For the present analysis, one participant retroactively withdrew consent for data usage. Then, 86 individuals had to be excluded due to missing or incomplete nutrition data. For 26 individuals, MRI data of the lumbar spine were missing or incomplete, resulting in a final sample of *N* = 297 participants in the main analysis. For the additional analysis of femoral BMAT, 150 individuals had to be excluded due to incomplete MRI data, resulting in a final sample of *N* = 163 individuals (Supplementary Fig. 1).

### Outcome assessment: MRI-derived bone marrow adipose tissue

MRI scans were performed on a 3 Tesla Magnetom Skyra (Siemens Healthineers, Erlangen, Germany) with a whole-body radiofrequency coil-matrix system. Details of the whole-body MRI protocol are provided elsewhere [16]. As part of the imaging protocol, a coronal 2-point Dixon T1-weighted VIBE sequence covering the torso was acquired. DIXON-based water and fat selective images were used to determine BMAT fat fraction within a region of interest as previously described [17]. Image analysis was performed using dedicated software (OsiriX 7.0, Pixmeo SARL, Bernex, Switzerland).

For vertebral BMAT, a single coronal image was used to manually quantify BMAT at L1 and L2 in the middle of the anterior–posterior diameter of the vertebral body. Cancellous bone was delineated on the fat image, copied to the water image, and cortical bone was excluded. Mean intensity values were then derived. BMAT at L1 and L2 were assessed separately and averaged. Similarly, for BMAT at the proximal femur, a single coronal slice covering the largest area of the femoral neck was analyzed. All cancellous bone was included and cortical bone excluded. Again, right and left femur were measured separately and averaged [17].

Additional MRI parameters included visceral adipose tissue (VAT) quantified from the femoral head to the diaphragm and hepatic fat content (HFC), which were derived as described previously [14].

### Exposure assessment: habitual dietary intake

Dietary intake was assessed using a combined approach, in which a self-administered food frequency questionnaire (FFQ) was supplemented with up to three 24-hour recalls completed within three months of the initial study visit (Supplementary Fig. 1) [18]. From these data, the probability of food consumption was derived and the typical quantity consumed on intake days was estimated based on the Bavarian Food Consumption Survey II [19]. Then, habitual diet was calculated as the product of consumption probability and amount. Food nutrient contents were derived by linking intake amounts to the German Nutrient Database (Bundeslebensmittelschlüssel, version 3.02) [14]. In our analysis, we considered total habitual energy intake, intake of energy-providing nutrients (fat, carbohydrates, protein, alcohol), and intake of micronutrients including calcium, phosphorus, and vitamin D.

### Covariate assessment

Information on lifestyle and risk factors, and medication intake was obtained through standardized face-to-face interviews and medical examinations, described in detail elsewhere [20].

In brief, BMI was calculated as weight (kg) divided by height squared (m²). Smoking status was self-reported and classified as never, former, or current smoker. Weekly leisure-time physical activity during summer and winter was self-reported with options “regularly more than or equal to two hours per week”, “regularly more than or equal to one, but less than two hours per week”, “less than one hour per week”, and “no exercise”. Responses for both seasons were combined into four categories “inactive”, “sporadically”, “regularly 1 h/w”, and “regularly 2 h/w” [21]. For subsequent analyses, physical activity was dichotomized into “irregular” (combining “inactive” and “sporadic”) and “regular ≥ 1 h/week” (combining “regularly 1 h/week” and “regularly 2 h/week”). Systolic and diastolic blood pressure were measured three times on the right arm after at least 5 min of rest, and the mean of the second and third measurements is reported. Hypertension was defined as blood pressure ≥ 140/90 mmHg or use of antihypertensive medication in participants aware of their condition. Glycemic status was classified into normoglycemia, prediabetes or diabetes based on either prior diagnosis of type 2 diabetes or an Oral Glucose Tolerance Test according to WHO guidelines [22]. Intake of antihypertensive, or lipid-lowering medication was self-reported.

Laboratory markers included total cholesterol, HDL, LDL, triglycerides, red and white blood cells, and hemoglobin, and were assessed by standard methods [14]. In women, postmenopausal status was defined as absence of menses for > 12 months, hysterectomy (with or without bilateral oophorectomy), or age > 60 years.

### Statistical analysis

Clinical characteristics, BMAT and dietary data are presented as mean and standard deviation or median and interquartile range for continuous variables and as count and percentage for categorical variables. Sex-specific differences were assessed by t-test or Χ² test, respectively. Energy-providing nutrients (fat, carbohydrates, protein, alcohol) were scaled to 1% of total energy intake, based on an energy value of 9 kcal/g for fat, 4 kcal/g for carbohydrates and protein, and 7 kcal/g for alcohol. Alcohol intake was log-transformed due to its skewed distribution.

Correlations between dietary parameters, MRI-derived BMAT, and blood parameters were evaluated using Spearman coefficients and visualized in sex-stratified scatter plots with linear smoothing. Potential non-linearity was assessed by visual inspection of LOESS-smoothed scatter plots.

Associations between habitual diet and BMAT were analyzed using linear regression with two adjustment sets as the main analysis: Model 1 was adjusted for age, sex, and physical activity (regular ≥ 1 h/week or irregular), and Model 2 additionally for BMI and diabetes status (normoglycemia, prediabetes, diabetes). These covariates were selected based on prior knowledge and clinical relevance. All models were calculated separately per nutrient (fat, carbohydrates, protein, alcohol, calcium, phosphorus, vitamin D). Results are presented as β coefficients with 95% confidence intervals (CI) and visualized in forest plots. As a supplementary analysis to evaluate the robustness of results for energy-providing nutrients, we additionally calculated linear regression-based substitution models [14]. As opposed to the main analysis where models were calculated separately per nutrient, the substitution model contains fat, protein, alcohol and total energy intake, i.e. all energy-providing nutrients except carbohydrates, and was adjusted for the two adjustment sets described above. Variables were scaled, so that the beta coefficients for fat, protein and alcohol denote an associated change in BMAT per 5% of total energy intake at the expense of carbohydrates, while the contributions from the respective other two energy-providing nutrients remain constant.

All regression models were fitted for the whole sample and stratified by sex. For analyses on the whole sample, formal tests for multiplicative interaction between nutrient intake and sex were additionally conducted. For the main analysis, P-values < 0.05 without correction for multiple testing were considered to indicate statistical significance, but a multiple testing correction (FDR) was additionally applied for additional interpretation R (version 4.3.3) was used for data handling, statistical modeling and creation of figures.

## Results

### Study sample

The final sample for the vertebral BMAT analysis included *N* = 297 participants (44.1% women) with a mean age of 56.1 ± 9.0 years and mean BMI of 28.0 ± 4.9 kg/m² (Table 1). Among women, 68.7% were postmenopausal. Overall, the prevalence of diabetes was 11.8% and of hypertension 34.0%. Participants included in the final sample did not differ significantly in their characteristics from those excluded due to missing vertebral BMAT or dietary data (Supplementary Table 1).

**Table 1:** Clinical characteristics of the study sample

	Whole sample	Men	Women	*p*-value
	*N* = 297	*N* = 166 (55.9%)	*N* = 131 (44.1%)	
Age, years	56.1 ± 9.0	56.1 ± 9.3	56.1 ± 8.6	0.961
Post-menopausal	-	-	90 (68.7%)	-
Body composition				
BMI, kg/m2	28.0 ± 4.9	28.3 ± 4.4	27.6 ± 5.5	0.196
VAT, l^#^	4.4 ± 2.7	5.6 ± 2.6	2.9 ± 2.1	< 0.001
HFC, %^##^	5.6 [2.9, 11.2]	7.5 [4.1, 14.1]	3.8 [2.3, 6.5]	< 0.001
BMAT, %				
Vertebral	54.7 ± 10.1	54.6 ± 9.5	54.8 ± 10.9	0.887
L1	52.9 ± 10.2	52.7 ± 9.8	53.2 ± 10.8	0.717
L2	56.5 ± 10.3	56.6 ± 9.5	56.5 ± 11.3	0.936
Femoral^###^	87.5 ± 5.1	89.8 ± 3.3	86.4 ± 5.5	< 0.001
Left hip	87.4 ± 5.2	89.8 ± 3.6	86.1 ± 5.4	< 0.001
Right hip	87.5 ± 5.2	89.5 ± 3.5	86.5 ± 5.6	< 0.001
Lipid profile				
Total Cholesterol, mg/dL	217.0 ± 35.9	215.5 ± 36.6	218.9 ± 35.1	0.422
HDL, mg/dL	62.4 ± 17.5	56.2 ± 14.9	70.4 ± 17.4	< 0.001
LDL, mg/dL	139.1 ± 33.2	141.3 ± 33.1	136.2 ± 33.3	0.192
Triglycerides, mg/dL	108.0 [77.0, 151.0]	120.6 [87.4, 181.1]	95.0 [69.5, 124.3]	< 0.001
Lipid-lowering medication	32 (10.8%)	16 (9.6%)	16 (12.2%)	0.602
Blood pressure				
Systolic BP, mmHg	120.0 ± 16.0	125.3 ± 15.1	113.3 ± 14.5	< 0.001
Diastolic BP, mmHg	75.0 ± 9.8	77.3 ± 10.1	72.1 ± 8.7	< 0.001
Hypertension	101 (34.0%)	66 (39.8%)	35 (26.7%)	0.026
Antihypertensive medication	78 (26.3%)	45 (27.1%)	33 (25.2%)	0.810
Diabetes				
Normoglycemia	186 (62.6%)	94 (56.6%)	92 (70.2%)	0.055
Prediabetes	76 (25.6%)	49 (29.5%)	27 (20.6%)	-
Diabetes	35 (11.8%)	23 (13.9%)	12 (9.2%)	-
Glucose-lowering medication	23 (7.7%)	13 (7.8%)	10 (7.6%)	1.000
Physical activity				
Regularly 2 h/w	85 (28.6%)	47 (28.3%)	38 (29.0%)	0.077
Regularly 1 h/w	97 (32.7%)	47 (28.3%)	50 (38.2%)	-
Sporadically	40 (13.5%)	21 (12.7%)	19 (14.5%)	-
Inactive	75 (25.3%)	51 (30.7%)	24 (18.3%)	-
Smoking				
Never-smoker	109 (36.7%)	53 (31.9%)	56 (42.7%)	0.063
Ex-smoker	131 (44.1%)	83 (50.0%)	48 (36.6%)	-
Smoker	57 (19.2%)	30 (18.1%)	27 (20.6%)	-
Hematologic parameters				
RBC, cells/pL	4.7 ± 0. 4	4.9 ± 0.4	4.5 ± 0.3	< 0.001
WBC, cells/nL	6.0 ± 1.7	6.0 ± 1.8	5.9 ± 1.6	0.582
Hemoglobin, g/L	143.5 ± 12.6	150.0 ± 10.6	135.2 ± 9.7	< 0.001

Values are given as mean ± SD or median [IQR] for continuous values and counts (percentage) for categorical values

*Abbreviations BMI * Body mass index, *VAT * visceral adipose tissue, *HFC * hepatic fat content, *HDL * high-density lipoprotein, *LDL * low-density lipoprotein, *BP * blood pressure, *RBC * red blood cells, *WBC * white blood cells

*P*-values from t-test or χ2 test, respectively

^#^*N* = 291. ^##^*N* = 294. ^###^*N* = 163

The femoral BMAT analysis included *N* = 163 participants (66.9% women) with a mean age of 57.5 ± 8.8 years. Among women, 73.4% were postmenopausal. Individuals excluded from the analysis due to missing femoral BMAT or dietary data had on average a more unfavourable cardiometabolic risk factor profile (Supplementary Table 2) as reported previously [13].

**Fig. 1:**
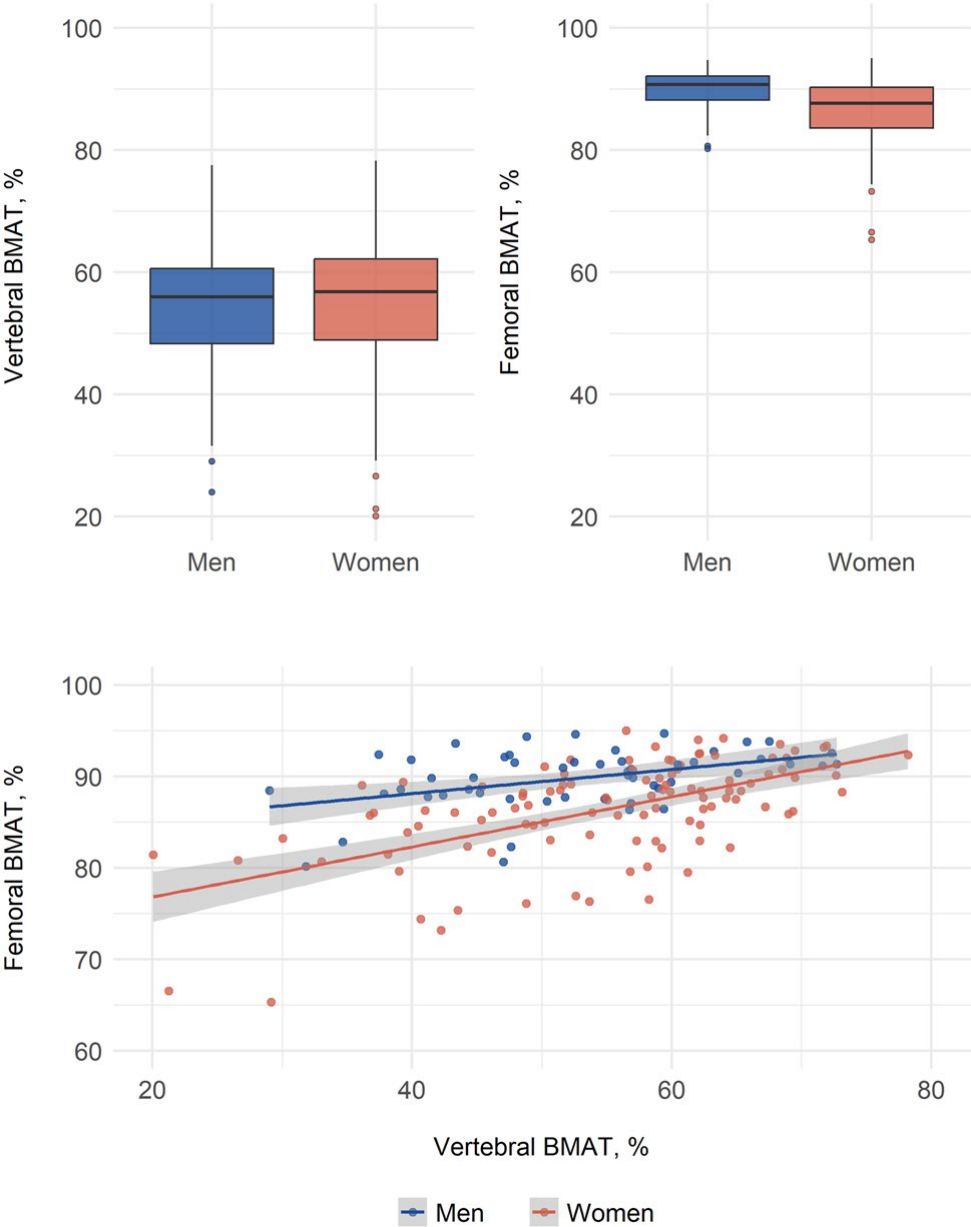
Distribution of vertebral and femoral BMAT. Sex-stratified boxplots and scatterplot with linear smoothing (*N* = 158 with both data)

Average vertebral BMAT was 54.6 ± 9.5% in men and 54.8 ± 10.9% in women. Average femoral BMAT was 89.8 ± 3.3% in men and 86.5 ± 5.5% in women (Fig. 1). Vertebral and femoral BMAT were positively correlated in both men (ρ = 0.36, *p* = 0.0096) and women (ρ = 0.54, *p* < 0.001).

Vertebral BMAT was not substantially correlated with red or white blood cell count, or hemoglobin (Supplementary Fig. 2). Only in women, higher femoral BMAT was correlated with lower white blood cell count (Supplementary Fig. 2).

Men had a total energy intake of 2059 ± 353 kcal/day, and women of 1555 ± 294 kcal/day. On average, 41.7% of energy was derived from carbohydrates, 38.0% from fat, 15.3% from protein, and 4.2% from alcohol, with significant sex differences. Average intake of phosphorus and vitamin D was higher in men, whereas calcium intake was higher in women (Table 2).

**Table 2:** Habitual diet of the study sample

	Whole sample	Men	Women	*p*-value
	*N* = 297	*N* = 166 (55.9%)	*N* = 131 (44.1%)	
Total energy intake, kcal/d	1837 ± 412	2059 ± 353	1555 ± 294	< 0.001
Fat, % of total EI	38.0 ± 3.5	37.4 ± 3.6	38.8 ± 3.3	0.001
Carbohydrates, % of total EI	41.7 ± 4.1	41.1 ± 4.3	42.5 ± 3.6	0.004
Protein, % of total EI	15.3 ± 1.7	14.9 ± 1.5	15.8 ± 1.7	< 0.001
Alcohol, % of total EI	4.2 ± 3.7	5.9 ± 3.8	2.0 ± 2.2	< 0.001
Calcium, mg/d	763.1 ± 205.4	744.1 ± 195.0	787.3 ± 216.2	0.072
Phosphorus, mg/d	1110.7 ± 263.9	1201.8 ± 252.1	995.4 ± 232.2	< 0.001
Vitamin D, µg/d	2.8 ± 1.1	3.1 ± 1.2	2.5 ± 0.8	< 0.001

Values are presented as means ± SD and *p*-values from t-test. *EI * Energy Intake

### Association between energy-providing nutrient intake and BMAT

**Fig. 2:**
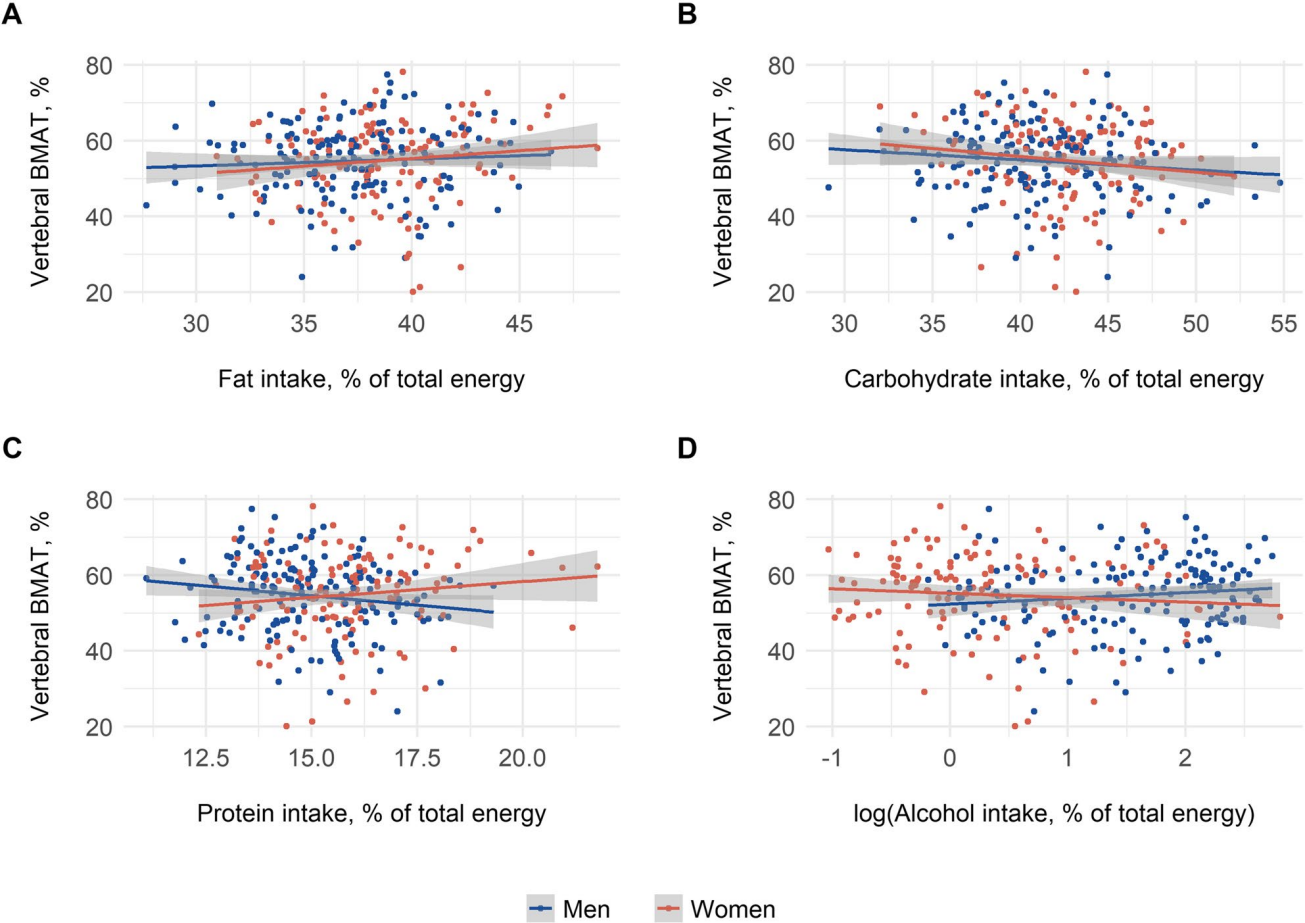
Correlation between energy-providing nutrients and vertebral BMAT. Sex-stratified scatterplots with linear smoothing showing (**A**) fat, **B** carbohydrate, **C** protein, and **D** alcohol intake

**Fig. 3:**
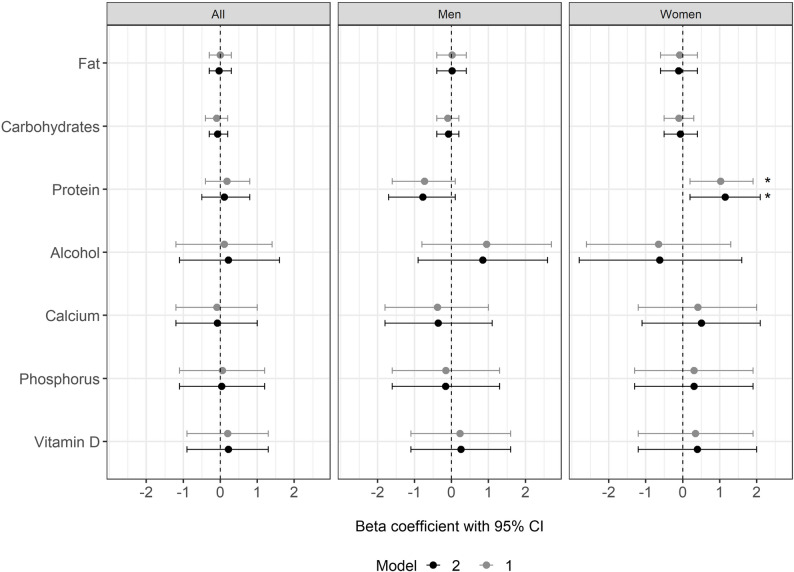
Association of habitual intake of energy-providing nutrients, calcium, phosphorus and vitamin D with vertebral BMAT. Displayed are beta coefficients with corresponding 95%CI from linear regression models with two adjustment sets: Model 1 was adjusted for age, sex, and physical activity; Model 2 additionally for BMI and diabetes status. Asterisks (*) indicate statistically significant associations (*p* < 0.05).

Fat intake (% of total energy) was not significantly correlated with vertebral BMAT in either men (ρ = 0.074, *p* = 0.34) or women (ρ = 0.13, *p* = 0.15, Fig. 2A). Consistently, in regression models both for the whole sample and sex-stratified, no significant associations or sex interactions across different adjustment sets were found (Fig. 3, Supplementary Table 3). Substitution models corroborated the non-significant associations (Supplementary Table 4).

Carbohydrate intake (% of total energy) showed no significant correlation with vertebral BMAT in men (ρ=-0.12, *p* = 0.11) or women (ρ=-0.14, *p* = 0.1, Fig. 2B), and regression models likewise indicated no associations or sex interactions (Fig. 3, Supplementary Table 3). Substitution models corroborated the non-significant associations (Supplementary Table 4).

Protein intake (% of total energy) showed opposing sex-specific associations with vertebral BMAT (Fig. 2C). In men, the association was negative and borderline significant (ρ = − 0.15, *p* = 0.057), whereas in women it was positive (ρ = 0.13, *p* = 0.14) and reached significance in regression models (β = 1.03, 95% CI [0.2, 1.9], Fig. 3, Supplementary Table 3). The interaction between sex and protein intake in the association with vertebral BMAT was statistically significant (*p* = 0.009, Supplementary Table 3). Substitution models corroborated the non-significant associations in men, and the positive association in women (Supplementary Table 4). While this finding was statistically significant in the main analysis, it was not significant after adjustment for multiple testing (Supplementary Table 3).

Alcohol intake (% of total energy) was not significantly correlated with vertebral BMAT in either men (ρ = 0.14, *p* = 0.08) or women (ρ=-0.087, *p* = 0.32, Fig. 2D), and no significant associations or sex interactions were observed in adjusted regression models (Fig. 3, Supplementary Table 3). Substitution models corroborated the non-significant associations (Supplementary Table 4).

Additional analyses of femoral BMAT showed no significant correlations with energy-providing nutrients apart from protein intake in men (ρ=-0.29, *p* = 0.036, Supplementary Fig. 3). Adjusted regression models did not reveal significant associations or sex interactions between femoral BMAT and any energy-providing nutrients (Supplementary Table 3, Supplementary Fig. 4).

Possible non-linear relationships were explored using LOESS smoothers, but no evidence of non-linearity was found (Supplementary Fig. 5).

### Association between micronutrients and BMAT

**Fig. 4:**
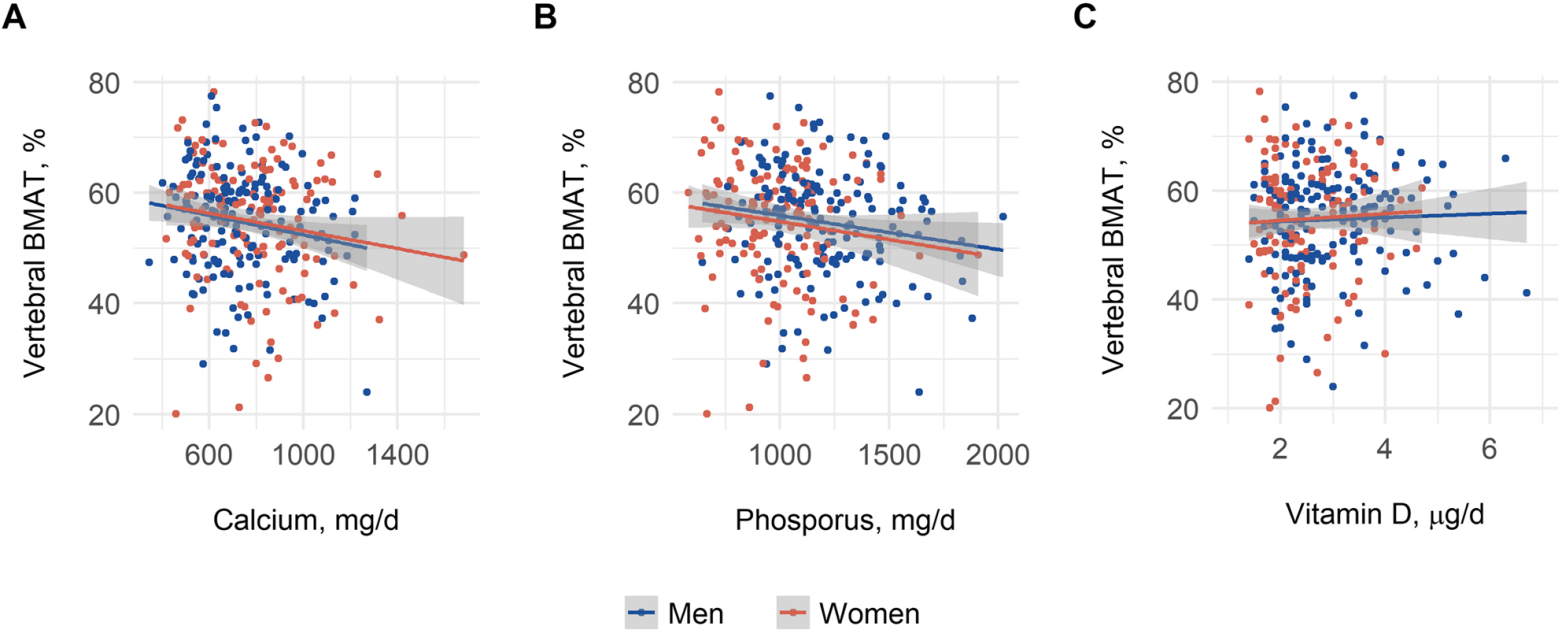
Correlation between micronutrients and vertebral BMAT. Sex-stratified scatterplots with linear smoothing showing (**A**) calcium, **B** phosphorus, and **C** vitamin D intake

Calcium intake was negatively correlated with vertebral BMAT in both men (ρ=-0.15, *p* = 0.047) and women (ρ=-0.18, *p* = 0.045, Fig. 4A). However, these effects were attenuated after adjustment in regression models for the total sample and in sex-specific analyses (Fig. 3, Supplementary Table 3).

Phosphorus intake showed a negative correlation with vertebral BMAT, which was significant in men (ρ=-0.17, *p* = 0.032) but not in women (ρ=-0.15, *p* = 0.081, Fig. 4B). After adjustment in regression models no significant effect was found (Fig. 3, Supplementary Table 3).

Vitamin D intake was not significantly correlated with vertebral BMAT in either men (ρ = 0.089, *p* = 0.26) or women (ρ = 0.025, *p* = 0.77, Fig. 4C), and no associations were observed after adjustment (Fig. 3, Supplementary Table 3).

In additional analyses on femoral BMAT, none of the correlations reached significance (Supplementary Fig. 6) and consistently, regression models showed no significant association (Supplementary Table 3, Supplementary Fig. 4). There were no significant interactions between sex and micronutrient intake in the association with neither vertebral nor femoral BMAT.

## Discussion

In this population-based sample, habitual dietary intake of energy-providing nutrients, calcium, phosphorus, or vitamin D showed no significant associations with MRI-derived bone marrow adipose tissue. A nominally significant association between protein intake and vertebral BMAT was observed in women in the main analysis; however, this finding should be interpreted cautiously given the exploratory nature of analyses. Although the observed findings may support potential site- and sex-specific differences, the lack of associations is in contrast to analyses on other fat depots such as skeletal muscles [13], VAT, SAT and liver [14].

BMAT differs molecularly and functionally from white, brown, and beige adipose tissue [23]. Unlike VAT or SAT, whose central roles in systemic energy homeostasis and as endocrine organs are established [24], the functions of BMAT are less understood. It is a local actor within the bone microenvironment, influencing bone remodeling and hematopoiesis [1]. Regarding its metabolic function, BMAT showed high basal glucose uptake but reduced insulin- and cold-stimulated glucose uptake [25], which may contribute to its ambiguous reaction to nutrient status. Changes in BMAT have been observed in response to extreme dietary regimes suggesting different functions in states with excessive or insufficient calorie intake [5, 26].

High fat diet has been found to increase BMAT both in short-term [27] and long-term mouse experiments [28] as well as in healthy humans [29]. Conversely, BMAT has also been shown to increase in individuals exposed to famine or starvation. This atypical response to energy deficit may reflect a possible role as an energy reservoir supporting hematopoiesis, which is an energetically demanding process [26].

A condition under which BMAT changes have been studied in detail is anorexia nervosa. Anorexia nervosa is a psychiatric disorder characterized by self-induced starvation, making this population a clinical model of chronic starvation. Compared to normal-weight, age-matched controls, women with anorexia nervosa had lower VAT and SAT but higher BMAT [30]. Cross-sectional results in recovered women suggest BMAT to decrease again in states of nutritional sufficiency [31].

Evidence on BMAT responses to obesity and weight loss remains inconsistent. Human studies report variable associations between obesity and BMAT, with some showing higher BMAT in individuals with obesity, whereas others find no difference or even lower levels [26]. The HELENA trial included 137 metabolically healthy adults with overweight or obesity, who underwent a randomized dietary intervention with calorie restriction for 12 weeks. MRI-derived BMAT significantly decreased with weight reduction and this effect was stronger with higher weight loss [32]. An 18-month randomized controlled trial with dietary intervention involving 138 adults demonstrated that weight loss can transiently reduce vertebral, but not femoral BMAT, with a more pronounced effect in younger participants highlighting that BMAT responses may vary with age and skeletal site [33]. Results from studies with patients who underwent bariatric surgery are inconsistent, with BMAT decreasing, increasing, or remaining unchanged depending on the surgery type [34–36].

The relation between weight loss and BMAT has also been studied in normal weight individuals. In a study of healthy adults who underwent a 10-day high-calorie intervention, followed by a 10-day fast, site- and sex-dependent BMAT dynamics were measured using proton magnetic resonance spectroscopy. Men showed significantly higher increase in vertebral BMAT compared to women, while women accumulated more femoral BMAT during fasting, supporting sex-specific differences in BMAT accumulation [29].

Overall, clinical studies remain limited and inconsistent, underscoring the complexity and heterogeneity of BMAT changes with metabolic perturbation [37]. In particular, BMAT responses to nutritional change appear to depend on how quickly energy intake and body weight change [29]. In our sample, neither of the extreme dietary regimes of high fat intake or caloric restriction were present. Participants showed average dietary patterns with a mean total energy intake of ~ 1800 kcal per day of which 38% stemmed from fat. Because the data were cross-sectional, we could not capture dynamic BMAT responses to changes in energy intake or body weight.

Micronutrients as the minerals calcium and phosphorus, as well as vitamin D, are essential for skeletal health and have been studied closely in relation to osteoporosis [10]. Vitamin D can be synthesized in the body and indirectly influences bone health by increasing intestinal calcium absorption and supporting bone mineralization. A deficiency of vitamin D in adults promotes low bone mass, muscle weakness, and a higher risk of fractures [38]. Calcium and phosphorus must be obtained from the diet and are the two basic mineral components of hydroxyapatite, the crystalline structure that gives bone its strength. Insufficient calcium intake can contribute to age-related bone loss and increased risk of osteoporosis. Dietary phosphorus deficiency is rare in humans due to its widespread presence in foods, a high phosphorus intake is generally well tolerated, with studies showing inconsistent evidence for adverse effects on bone health [10]. Direct evidence linking calcium, phosphorus, or vitamin D intake to BMAT is limited and no human studies have directly investigated the effects. In our sample, we did not observe significant associations between dietary calcium, phosphorus, or vitamin D intake and BMAT.

We observed that protein intake was differently associated with vertebral BMAT in men and women, a pattern not seen at the femoral site. The site-specific pattern may reflect genuine regional differences in BMAT behavior. This finding is consistent with prior work showing that proximal and distal skeletal sites respond differently to metabolic changes [39]. The sex-specific association could be incidental or partly driven by reverse causality, but it may also signal true biological differences, as women differ in dietary patterns, osteoporosis risk, and prior studies highlight sex-dependent variation in BMAT composition and in responses to nutritional and hormonal factors [7, 40, 41]. However, since this finding was not statistically significant after correction for multiple testing, it could be a chance finding that needs to be interpreted with caution and needs further corroboration in other studies.

We also observed site- and sex-specific trends in the association between BMAT and hematopoietic markers. Vertebral BMAT showed an inverse relationship with RBC, WBC, and hemoglobin in men and women, though these associations did not reach statistical significance. Interestingly, femoral BMAT was significantly negatively associated with WBC in women. Previous studies have reported inconsistent findings. In 137 adults with overweight or obesity, no significant associations were found between RBC, WBC, and vertebral BMAT, whereas in a study of 89 premenopausal women (including 35 with anorexia nervosa), negative associations were reported between femoral BMAT and WBC, RBC, and between vertebral BMAT and WBC. This further supports site-specific differences and suggests that hormonal changes with aging, particularly for women during menopause, as well as higher hematopoietic demands in premenopausal women due to menstrual blood loss, may help explain the observed sex differences [42].

It is important to note the methodological strengths of our study. MRI as an established and robust imaging modality allows the accurate and non-invasive quantification of BMAT. BMAT assessment at two skeletal sites and including hematological parameters allowed us to explore regional differences and its potential links to hematopoiesis. Finally, in stratified analyses we were able to explore sex-specific associations that have been suggested in experimental models. Importantly, this work is among the few studies examining BMAT in a sample from a population-based cohort rather than in patient collectives exposed to extreme nutritional states. This allowed us to evaluate how BMAT relates to everyday dietary patterns in adults. Dietary intake was assessed with both a food-frequency questionnaire and 24-hour recalls, offering complementary information on habitual and recent nutrition and reducing single-method bias.

Several limitations should be considered. First, the sample size was relatively small, which limited our ability to further examine the relationship between dietary patterns and BMAT in different subgroups. In particular, we were unable to perform sufficiently powered stratified analyses by sex, age, BMI, and menopausal status, although these factors may substantially influence BMAT and its association with nutrition. The limited sample size may also have reduced the power to detect potential non-linear associations or interaction effects. Larger population-based cohorts, such as the German National Cohort (NAKO), which includes both detailed whole-body imaging and nutritional assessment, will allow these effects to be examined in greater detail. Second, although BMAT is a dynamic adipose tissue depot that is suggested to respond rapidly and reversibly to nutrient changes, our study is cross-sectional. As a result, we cannot capture short- or long-term BMAT dynamics, nor can we infer causality or temporal relationships between diet and BMAT. Third, despite using validated dietary assessment methods, measurement errors due to self-reported habitual diet have to be considered. In addition, BMAT likely reflects long-term dietary patterns, whereas dietary intake was assessed over a limited time period. This temporal mismatch may have attenuated potential associations between diet and BMAT.

In conclusion, this analysis of a sample from a population-based cohort did not reveal a consistent pattern of significant associations between habitual dietary intake and BMAT overall. Although some exploratory analyses indicated possible site- and sex-specific differences, these findings should be interpreted with caution given the largely null results. Nevertheless, they align with the concept that BMAT is a heterogeneous fat depot whose regulation may involve complex interactions between local factors, systemic metabolism, and hormonal status. Further investigations in larger cohorts are needed to better understand potential sex- and age-related effects.

## Supplementary Information

Supplementary Material 1.

## Data Availability

The datasets analyzed during the current study are not publicly available due to national data protection laws, since the informed consent given by KORA study participants does not cover data posting in public databases. Data are available upon request by means of a project agreement from KORA. Requests should be sent to kora.passt@helmholtz-munich.de and are subject to approval by the KORA Board. Analysis codes are available from the authors upon reasonable request.
