# Supplementary material for: Association of habitual diet and bone marrow adipose tissue – magnetic resonance imaging in a population-based sample
Source: Nutr J. 2026 Jul 8;25:74. doi: 10.1186/s12937-026-01365-z (PMC13343689; doi:10.1186/s12937-026-01365-z)

**Supplementary material to**

**Association of habitual diet and bone marrow adipose tissue – magnetic resonance imaging in a population-based sample**

*Short title: Habitual diet and bone marrow fat*

Elena Grune^1,2,3^, Dunja Hasic^4^, Christopher L. Schlett^1^, Fabian Bamberg^1^, Annette Peters^2,3,5,6^, Nina Wawro^2,7^, Jakob Linseisen^7^, Susanne Rospleszcz^1^, Marc-Nicolas v. Itter^1^

**Supplementary Table 1:** Characteristics of participants excluded from the sample for the analysis of vertebral BMAT and of participants included in the sample for the analysis of vertebral BMAT.

|  | Excluded | Included | p-value |
| --- | --- | --- | --- |
|  | N = 102 | N = 297 |  |
| Age, years | 56.1 ± 9.0 | 56.1 ± 9.0 | 0.395 |
| Men | 64 (62.7%) | 166 (55.9%) | 0.275 |
| Women | 38 (37.3%) | 131 (44.1%) | - |
| Post-menopause (% of women) | 23 (60.5%) | 90 (68.7) | 0.455 |
| **Body composition** |  |  |  |
| BMI, kg/m^2^ | 28.6 ± 4.9 | 28.0 ± 4.9 | 0.269 |
| VAT | 4.9 ± 2.6 | 4.4 ± 2.7 | 0.104 |
| HFC, % | 6.2 [3.0, 13.4] | 5.6 [2.9, 11.2] | 0.306 |
| **Lipid profile** |  |  |  |
| Total Cholesterol, mg/dL | 220.4 ± 37.4 | 217.0 ± 35.9 | 0.424 |
| HDL, mg/dL | 60.3 ± 18.1 | 62.4 ± 17.5 | 0.293 |
| LDL, mg/dL | 141.2 ± 32.1 | 139.1 ± 33.2 | 0.580 |
| Triglycerides, mg/dL | 109.1 [76.7, 173.8] | 108.0 [77.0, 151.0] | 0.312 |
| Lipid-lowering medication | 11 (10.8%) | 32 (10.8%) | 1 |
| **Blood pressure** |  |  |  |
| Systolic BP, mmHg | 122.7 ± 18.7 | 120.0 ± 16.0 | 0.156 |
| Diastolic BP, mmHg | 76.2 ± 10.5 | 75.0 ± 9.8 | 0.289 |
| Hypertension | 35 (34.3%) | 101 (34.0%) | 1 |
| Antihypertensive medication | 24 (23.5%) | 78 (26.3%) | 0.679 |
| **Diabetes** |  |  |  |
| Normoglycemia | 56 (54.9%) | 186 (62.6%) | 0.183 |
| Prediabetes | 27 (26.5%) | 76 (25.6%) | - |
| Diabetes | 19 (18.6%) | 35 (11.8%) | - |
| Glucose-lowering medication | 9 (8.8%) | 23 (7.7%) | 0.893 |
| **Physical activity** |  |  |  |
| Regularly, 2h/w | 29 (28.4%) | 85 (28.6%) | 0.511 |
| Regularly, 1h/w | 26 (25.5%) | 97 (32.7%) | - |
| Sporadically | 17 (16.7%) | 40 (13.5%) | - |
| Inactive | 30 (29.4%) | 75 (25.3%) | - |
| **Smoking** |  |  |  |
| Never-smoker | 36 (35.3%) | 109 (36.7%) | 0.766 |
| Ex-smoker | 43 (42.2%) | 131 (44.1%) | - |
| Smoker | 23 (22.5%) | 57 (19.2%) | - |
| **Hematologic parameters** |  |  |  |
| RBC, cells/pL | 4.7 ± 0.4 | 4.7 ± 0.4 | 0.483 |
| WBC, cells/nL | 6.1 ± 1.7 | 6.0 ± 1.7 | 0.489 |
| Hemoglobin, g/L | 144.8 ± 11.6 | 143.5 ± 12.6 | 0.330 |

Values are given as mean ± SD or median [IQR] for continuous values and counts (percentage) for categorical values. P-values from t-test or χ2 test, respectively. Abbreviations. BMI: Body mass index, VAT: visceral adipose tissue, HFC: hepatic fat content, HDL: high-density lipoprotein, LDL: low-density lipoprotein, BP: blood pressure, RBC: red blood cells, WBC: white blood cells.

**Supplementary Table 2:** Characteristics of participants excluded from the sample for the analysis of femoral BMAT and of participants included in the sample for the analysis of femoral BMAT.

|  | Excluded | Included | p-value |
| --- | --- | --- | --- |
|  | N = 236 | N = 163 |  |
| Age, years | 56.1 ± 9.0 | 57.5 ± 8.8 | 0.035 |
| Men | 176 (74.6%) | 54 (33.1%) | <0.001 |
| Women | 60 (25.4%) | 109 (66.9%) | - |
| Post-menopause (% of women) | 33 (55.0%) | 80 (73.4%) | 0.024 |
| **Body composition** |  |  |  |
| BMI, kg/m^2^ | 28.8 ± 5.0 | 27.1 ± 4.6 | <0.001 |
| VAT | 5.3 ± 2.6 | 3.5 ± 2.4 | <0.001 |
| HFC, % | 6.8 [3.5, 14.1] | 4.4 [2.4, 8.8] | <0.001 |
| **Lipid profile** |  |  |  |
| Total Cholesterol, mg/dL | 218.7 ± 37.3 | 216.7 ± 34.9 | 0.585 |
| HDL, mg/dL | 57.4 ± 16.0 | 68.5 ± 17.9 | <0.001 |
| LDL, mg/dL | 142.9 ± 32.9 | 134.8 ± 32.3 | 0.016 |
| Triglycerides, md/dL | 119.7 [85.0, 176.6] | 95.0 [71.6, 132.5] | <0.001 |
| Lipid-lowering medication | 19 (8.1%) | 24 (14.7%) | 0.051 |
| **Blood pressure** |  |  |  |
| Systolic BP, mmHg | 122.9 ± 16.6 | 117.4 ± 16.5 | 0.001 |
| Diastolic BP, mmHg | 76.4 ± 10.0 | 73.7 ± 9.9 | 0.010 |
| Hypertension | 83 (35.2%) | 53 (32.5%) | 0.658 |
| Antihypertensive medication | 54 (22.9%) | 48 (29.4%) | 0.173 |
| **Diabetes** |  |  |  |
| Normoglycemia | 126 (53.4%) | 116 (71.2%) | 0.002 |
| Prediabetes | 73 (30.9%) | 30 (18.4%) | - |
| Diabetes | 37 (15.7%) | 17 (10.4%) | - |
| Glucose-lowering medication | 19 (8.1%) | 13 (8.0%) | 1 |
| **Physical activity** |  |  |  |
| Regularly 2h/w | 60 (25.4%) | 54 (33.1%) | 0.178 |
| Regularly 1h/w | 70 (29.7%) | 53 (32.5%) | - |
| Sporadically | 37 (15.7%) | 20 (12.3%) | - |
| Inactive | 69 (29.2%) | 36 (22.1%) | - |
| **Smoking** |  |  |  |
| Never-smoker | 75 (31.8%) | 70 (42.9%) | 0.056 |
| Ex-smoker | 113 (47.9%) | 61 (37.4%) | - |
| Smoker | 48 (20.3%) | 32 (19.6%) | - |
| **Hematologic parameters** |  |  |  |
| RBC, cells/pL | 4.8 ± 0.4 | 4.6 ± 0.4 | <0.001 |
| WBC, cells/nL | 6.1 ± 1.9 | 5.9 ± 1.5 | 0.359 |
| Hemoglobin, g/L | 146.4± 11.6 | 140.1 ± 12.6 | <0.001 |

Values are given as mean ± SD or median [IQR] for continuous values and counts (percentage) for categorical values. P-values from t-test or χ2 test, respectively. Abbreviations. BMI: Body mass index, VAT: visceral adipose tissue, HFC: hepatic fat content, HDL: high-density lipoprotein, LDL: low-density lipoprotein, BP: blood pressure, RBC: red blood cells, WBC: white blood cells.

**Supplementary Table 3:** Association of habitual intake of energy-providing nutrients, calcium, phosphorus and vitamin D with vertebral and femoral BMAT (corresponds to main Figure 3). Asterisks (*) indicate statistically significant associations (p < 0.05).

|  |  | Outcome vertebral BMAT, % | | | | Outcome femoral BMAT, % | | | |
| --- | --- | --- | --- | --- | --- | --- | --- | --- | --- |
|  |  | β | 95%-CI | p-value | Adj p-value | β | 95%-CI | p-value | Adj p-value |
| **Fat, per 1% of total energy intake** | | | | | | | | | |
| All | Model 1 | 0 | [-0.3, 0.3] | 0.979 | 0.979 | -0.14 | [-0.4, 0.1] | 0.273 | 0.979 |
|  | Model 2 | -0.03 | [-0.3, 0.3] | 0.867 | 0.979 | -0.05 | [-0.3, 0.2] | 0.656 | 0.979 |
| Multiplicative sex interaction | | | | 0.761 | 0.979 |  | | 0.526 | 0.979 |
| Men | Model 1 | 0.02 | [-0.4, 0.4] | 0.926 | 0.979 | -0.27 | [-0.6, 0.0] | 0.052 | 0.979 |
|  | Model 2 | 0.02 | [-0.4, 0.4] | 0.924 | 0.979 | -0.24 | [-0.5, 0.1] | 0.109 | 0.979 |
| Women | Model 1 | -0.08 | [-0.6, 0.4] | 0.759 | 0.979 | -0.11 | [-0.5, 0.2] | 0.511 | 0.979 |
|  | Model 2 | -0.11 | [-0.6, 0.4] | 0.652 | 0.979 | -0.04 | [-0.4, 0.3] | 0.786 | 0.979 |
| **Carbohydrates, per 1% of total energy intake** | | | | | | | | | |
| All | Model 1 | -0.10 | [-0.4, 0.2] | 0.438 | 0.979 | -0.10 | [-0.4, 0.2] | 0.438 | 0.979 |
|  | Model 2 | -0.07 | [-0.3, 0.2] | 0.590 | 0.979 | 0.03 | [-0.2, 0.2] | 0.771 | 0.979 |
| Multiplicative sex interaction | | | | 0.931 | 0.979 |  | | 0.755 | 0.979 |
| Men | Model 1 | -0.10 | [-0.4, 0.2] | 0.522 | 0.979 | 0.15 | [-0.0, 0.3] | 0.123 | 0.979 |
|  | Model 2 | -0.08 | [-0.4, 0.2] | 0.607 | 0.979 | 0.09 | [-0.1, 0.3] | 0.387 | 0.979 |
| Women | Model 1 | -0.10 | [-0.5, 0.3] | 0.658 | 0.979 | 0.09 | [-0.2, 0.4] | 0.572 | 0.979 |
|  | Model 2 | -0.06 | [-0.5, 0.4] | 0.783 | 0.979 | 0.03 | [-0.3, 0.3] | 0.865 | 0.979 |
| **Protein, per 1% of total energy intake** | | | | | | | | | |
| All | Model 1 | 0.18 | [-0.4, 0.8] | 0.574 | 0.979 | -0.36 | [-0.8, 0.1] | 0.133 | 0.979 |
|  | Model 2 | 0.11 | [-0.5, 0.8] | 0.738 | 0.979 | -0.02 | [-0.5, 0.5] | 0.928 | 0.979 |
| Multiplicative sex interaction | | | | 0.009* | 0.653 |  | | 0.679 | 0.979 |
| Men | Model 1 | -0.73 | [-1.6, 0.1] | 0.096 | 0.979 | -0.54 | [-1.2, 0.1] | 0.079 | 0.979 |
|  | Model 2 | -0.77 | [-1.7, 0.1] | 0.092 | 0.979 | -0.30 | [-0.9, 0.4] | 0.359 | 0.979 |
| Women | Model 1 | 1.03 | [0.2, 1.9] | 0.020* | 0.653 | -0.29 | [-0.9, 0.3] | 0.353 | 0.979 |
|  | Model 2 | 1.15 | [0.2, 2.1] | 0.017* | 0.653 | 0.14 | [-0.5, 0.8] | 0.662 | 0.979 |
| **Alcohol, per log(1% of total energy intake)** | | | | | | | | | |
| All | Model 1 | 0.11 | [-1.2, 1.4] | 0.861 | 0.979 | 0.65 | [-0.4, 1.7] | 0.213 | 0.979 |
|  | Model 2 | 0.23 | [-1.1, 1.6] | 0.742 | 0.979 | 0.02 | [-1.0, 1.0] | 0.965 | 0.979 |
| Multiplicative sex interaction | | | | 0.464 | 0.979 |  | | 0.824 | 0.979 |
| Men | Model 1 | 0.95 | [-0.8, 2.7] | 0.273 | 0.979 | 0.19 | [-1.1, 1.5] | 0.770 | 0.979 |
|  | Model 2 | 0.85 | [-0.9, 2.6] | 0.343 | 0.979 | 0.31 | [-1.0, 1.6] | 0.630 | 0.979 |
| Women | Model 1 | -0.65 | [-2.6, 1.3] | 0.504 | 0.979 | 0.96 | [-0.4, 2.3] | 0.165 | 0.979 |
|  | Model 2 | -0.62 | [-2.8, 1.6] | 0.575 | 0.979 | -0.10 | [-1.5, 1.3] | 0.884 | 0.979 |
| **Calcium, per SD** | | | | | | | | | |
| All | Model 1 | -0.09 | [-1.2, 1.0] | 0.874 | 0.979 | 0.24 | [-0.6, 1.0] | 0.553 | 0.979 |
|  | Model 2 | -0.08 | [-1.2, 1.0] | 0.884 | 0.979 | 0.1 | [-0.7, 0.9] | 0.799 | 0.979 |
| Multiplicative sex interaction | | | | 0.809 | 0.979 |  | | 0.278 | 0.979 |
| Men | Model 1 | -0.38 | [-1.8, 1.0] | 0.600 | 0.979 | 0.3 | [-0.8, 1.4] | 0.586 | 0.979 |
|  | Model 2 | -0.36 | [-1.8, 1.1] | 0.623 | 0.979 | 0.44 | [-0.6, 1.5] | 0.417 | 0.979 |
| Women | Model 1 | 0.41 | [-1.2, 2.0] | 0.613 | 0.979 | 0.28 | [-0.8, 1.3] | 0.595 | 0.979 |
|  | Model 2 | 0.51 | [-1.1, 2.1] | 0.533 | 0.979 | 0.09 | [-0.9, 1.1] | 0.860 | 0.979 |
| **Phosphorus, per SD** | | | | | | | | | |
| All | Model 1 | 0.06 | [-1.1, 1.2] | 0.927 | 0.979 | 0.15 | [-0.7, 1.0] | 0.724 | 0.979 |
|  | Model 2 | 0.04 | [-1.1, 1.2] | 0.949 | 0.979 | 0.06 | [-0.8, 0.9] | 0.890 | 0.979 |
| Multiplicative sex interaction | | | | 0.942 | 0.979 |  | | 0.311 | 0.979 |
| Men | Model 1 | -0.15 | [-1.6, 1.3] | 0.835 | 0.979 | 0.26 | [-0.7, 1.2] | 0.588 | 0.979 |
|  | Model 2 | -0.16 | [-1.6, 1.3] | 0.824 | 0.979 | 0.39 | [-0.6, 1.3] | 0.417 | 0.979 |
| Women | Model 1 | 0.31 | [-1.3, 1.9] | 0.709 | 0.979 | 0.09 | [-1.0, 1.1] | 0.872 | 0.979 |
|  | Model 2 | 0.31 | [-1.3, 1.9] | 0.710 | 0.979 | -0.04 | [-1.0, 0.9] | 0.938 | 0.979 |
| **Vitamin D, per SD** | | | | | | | | | |
| All | Model 1 | 0.2 | [-0.9, 1.3] | 0.717 | 0.979 | -0.17 | [-1.0, 0.6] | 0.682 | 0.979 |
|  | Model 2 | 0.22 | [-0.9, 1.3] | 0.687 | 0.979 | -0.19 | [-1.0, 0.6] | 0.630 | 0.979 |
| Multiplicative sex interaction | | | | 0.474 | 0.979 |  | | 0.682 | 0.979 |
| Men | Model 1 | 0.23 | [-1.1, 1.6] | 0.739 | 0.979 | -0.20 | [-1.0, 0.6] | 0.634 | 0.979 |
|  | Model 2 | 0.26 | [-1.1, 1.6] | 0.713 | 0.979 | -0.18 | [-1.0, 0.6] | 0.659 | 0.979 |
| Women | Model 1 | 0.35 | [-1.2, 1.9] | 0.659 | 0.979 | -0.08 | [-1.1, 0.9] | 0.870 | 0.979 |
|  | Model 2 | 0.4 | [-1.2, 2.0] | 0.622 | 0.979 | -0.02 | [-1.0, 0.9] | 0.973 | 0.979 |

SD: Standard Deviation. Standard deviations were: Calcium 205.4 mg (men: 195.0 mg, women: 216.2 mg), Phosporus 263.9 mg (men: 252.1 mg, women: 232.2 mg), Vitamin D 1.1 μg (men: 1.2 μg, women: 0.8 μg). Adjusted p-values were FDR adjusted for n=98 tests.

**Supplementary Table 4:** Substitution model: Association of an isocaloric replacement of carbohydrates with fat, protein or alcohol with vertebral and femoral BMAT. Asterisks (*) indicate statistically significant associations (p < 0.05).

|  |  | Outcome vertebral BMAT, % | | | | Outcome femoral BMAT, % | | | |
| --- | --- | --- | --- | --- | --- | --- | --- | --- | --- |
|  |  | β | 95%-CI | p-value | p-value adj | β | 95%-CI | p-value | p-value adj |
| **Fat, per substitution of 5% of total energy intake at expense of carbohydrates** | | | | | | | | | |
| All | Model 1 | -0.05 | [-1.7, 1.6] | 0.952 | 0.987 | -0.43 | [-1.7, 0.8] | 0.495 | 0.927 |
|  | Model 2 | -0.15 | [-1.8, 1.5] | 0.860 | 0.987 | -0.25 | [-1.5, 1.0] | 0.680 | 0.987 |
| Men | Model 1 | 1.22 | [-1.0, 3.4] | 0.275 | 0.882 | -0.99 | [-2.5, 0.5] | 0.199 | 0.882 |
|  | Model 2 | 1.18 | [-1.0, 3.4] | 0.297 | 0.882 | -0.97 | [-2.6, 0.6] | 0.224 | 0.882 |
| Women | Model 1 | -1.00 | [-3.5, 1.5] | 0.436 | 0.912 | -0.31 | [-2.1, 1.4] | 0.730 | 0.987 |
|  | Model 2 | -1.11 | [-3.7, 1.4] | 0.389 | 0.882 | -0.27 | [-1.9, 1.4] | 0.749 | 0.987 |
| **Protein, per substitution of 5% of total energy intake at expense of carbohydrates** | | | | | | | | | |
| All | Model 1 | 1.06 | [-2.3, 4.5] | 0.541 | 0.927 | -1.49 | [-4.0, 1.0] | 0.231 | 0.882 |
|  | Model 2 | 0.73 | [-2.8, 4.2] | 0.686 | 0.987 | 0.02 | [-2.5, 2.5] | 0.985 | 0.987 |
| Men | Model 1 | -4.34 | [-9.3, 0.6] | 0.084 | 0.756 | -1.51 | [-5.0, 2.0] | 0.392 | 0.882 |
|  | Model 2 | -4.63 | [-9.7, 0.5] | 0.075 | 0.756 | -0.17 | [-3.9, 3.5] | 0.927 | 0.987 |
| Women | Model 1 | 5.01 | [0.4, 9.6] | 0.032* | 0.576 | -1.20 | [-4.4, 2.0] | 0.456 | 0.912 |
|  | Model 2 | 5.67 | [0.9, 10.5] | 0.021* | 0.576 | 0.58 | [-2.6, 3.7] | 0.717 | 0.987 |
| **Alcohol, per substitution of 5% of total energy intake at expense of carbohydrates** | | | | | | | | | |
| All | Model 1 | 0.18 | [-1.2, 1.5] | 0.798 | 0.987 | 0.52 | [-0.5, 1.6] | 0.324 | 0.882 |
|  | Model 2 | 0.22 | [-1.2, 1.6] | 0.759 | 0.987 | -0.01 | [-1.0, 1.0] | 0.987 | 0.987 |
| Men | Model 1 | 0.92 | [-0.9, 2.7] | 0.314 | 0.882 | 0.01 | [-1.3, 1.3] | 0.983 | 0.987 |
|  | Model 2 | 0.84 | [-1.0, 2.7] | 0.370 | 0.882 | 0.04 | [-1.3, 1.4] | 0.956 | 0.987 |
| Women | Model 1 | -0.66 | [-2.7, 1.4] | 0.523 | 0.927 | 0.83 | [-0.6, 2.3] | 0.255 | 0.882 |
|  | Model 2 | -1.03 | [-3.3, 1.2] | 0.369 | 0.882 | -0.21 | [-1.7, 1.3] | 0.781 | 0.987 |

Model 1 was adjusted for age, sex, and physical activity (regular ≥ 1 h/week or irregular), and Model 2 additionally for BMI and diabetes status (normoglycemia, prediabetes, diabetes). Adjusted p-values were FDR adjusted for n=36 tests.

**Supplementary Figure 1: Participant flowchart (panel A) and timeline of examinations (panel B).**


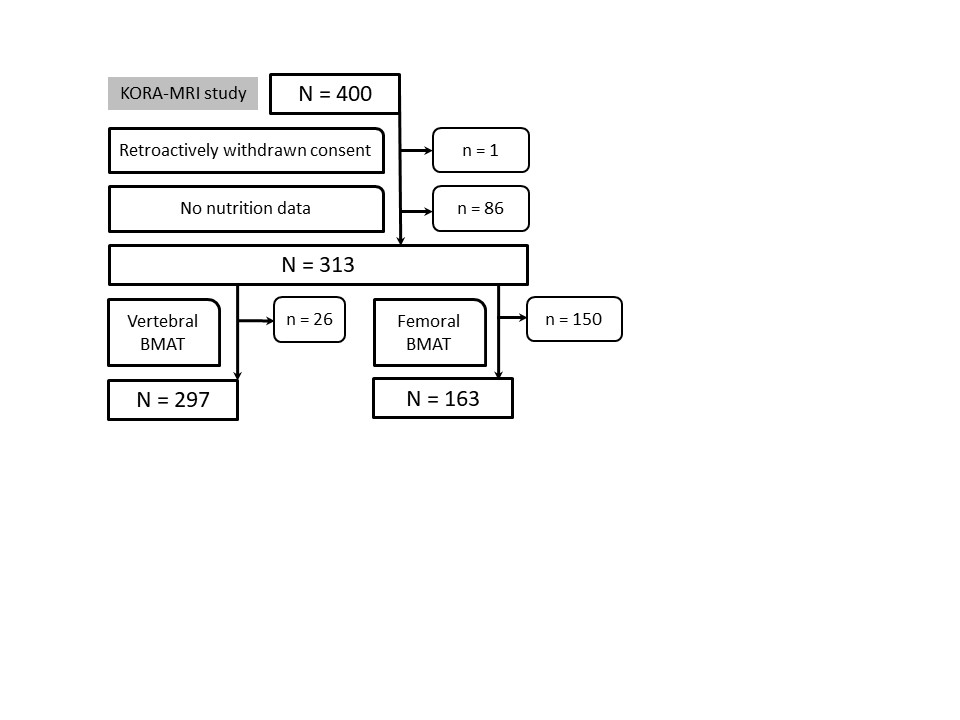
A.


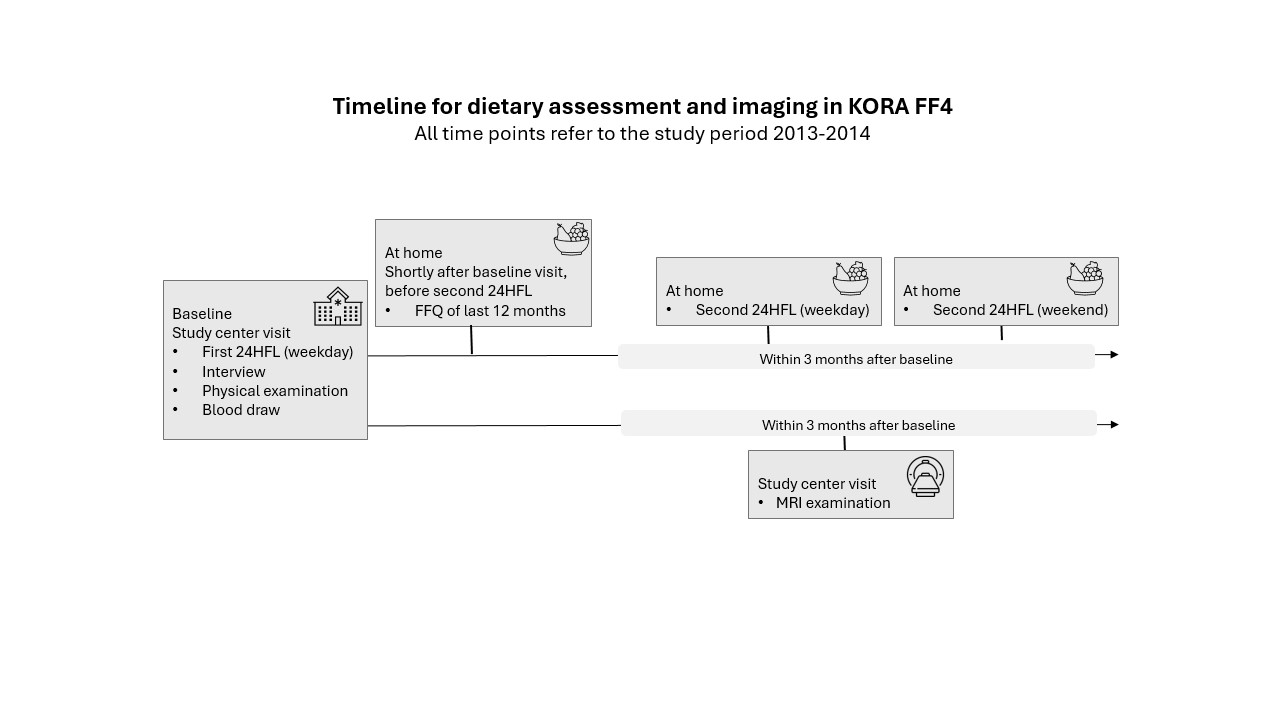


B.

**Supplementary Figure 2.** **Correlation between hematologic parameters and vertebral and femoral BMAT.** Sex-stratified scatterplots with linear smoothing
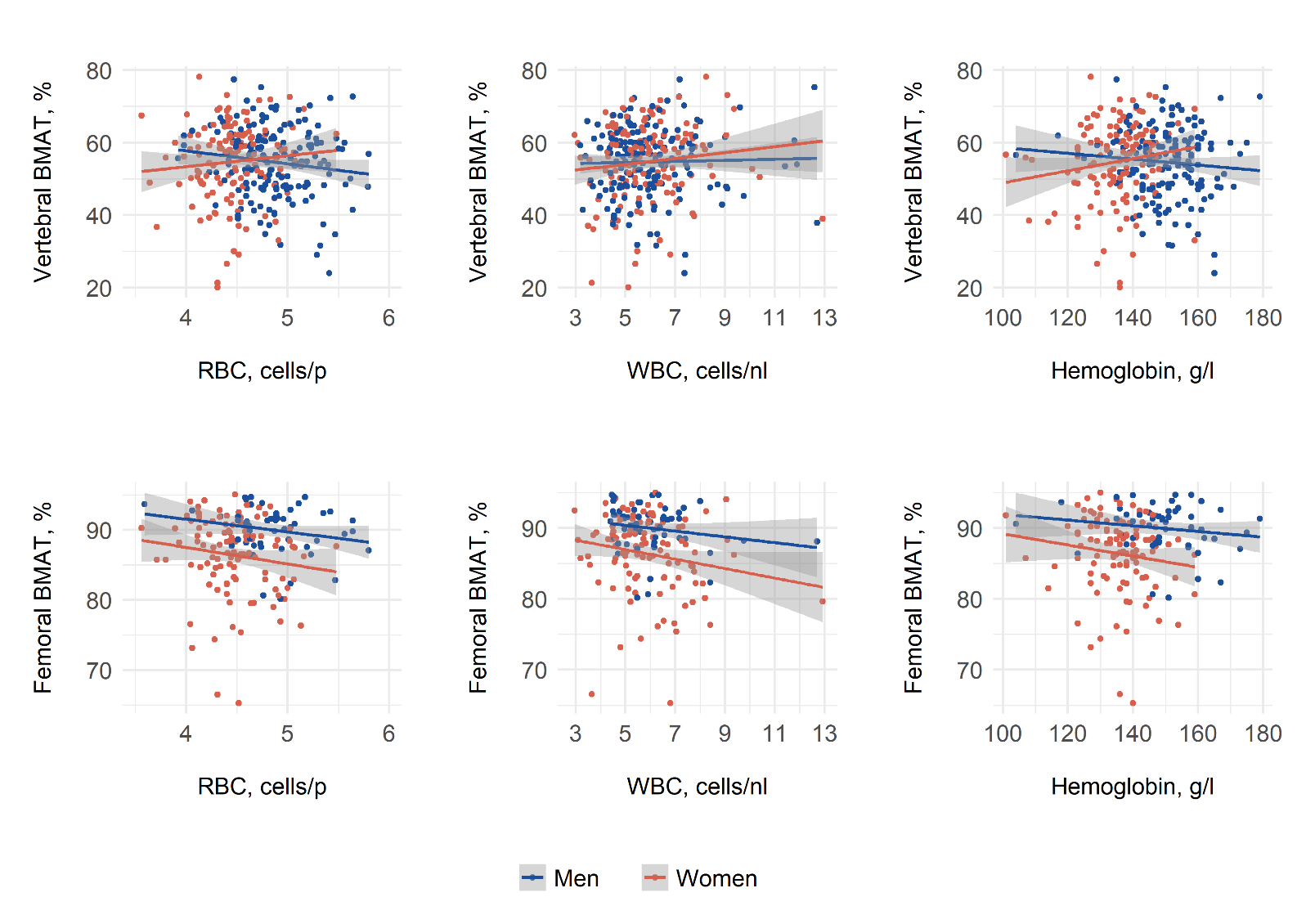
.

**Supplementary Figure 3: Correlation between energy-providing nutrients and femoral BMAT.** Sex-stratified scatterplots with linear smoothing.


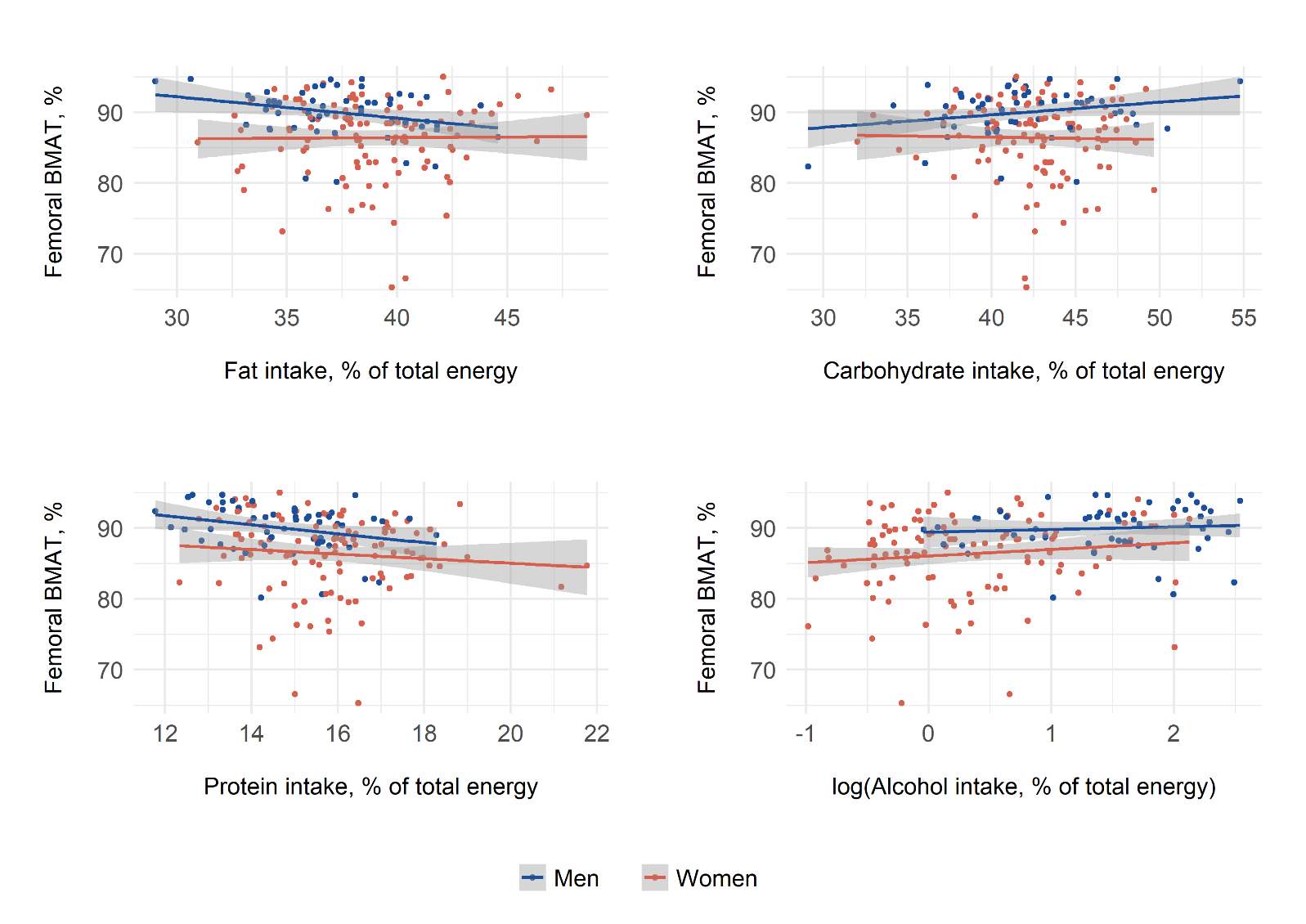


**
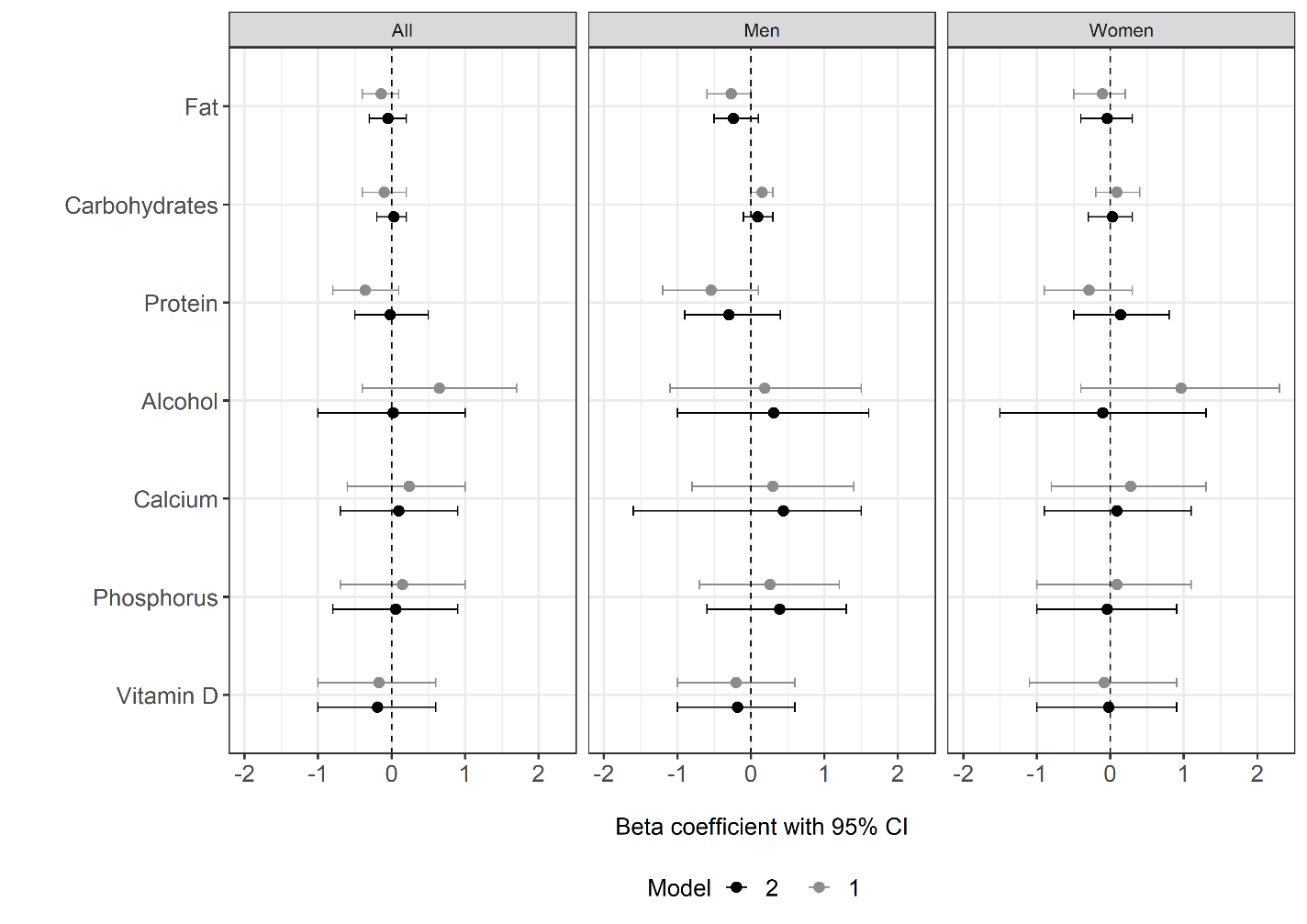
Supplementary Figure 4: Association of habitual intake of energy-providing nutrients, calcium, phosphorus and vitamin D with femoral BMAT.** Displayed are beta coefficients with corresponding 95%CI from linear regression models with two adjustment sets: Model 1 was adjusted for age, sex, and physical activity; Model 2 additionally for BMI and diabetes status.

**Supplementary Figure 5:** **Non-linearity of correlations of habitual intake of energy-providing nutrients with vertebral and femoral BMAT.** Sex-stratified scatterplots with LOESS smoothing.


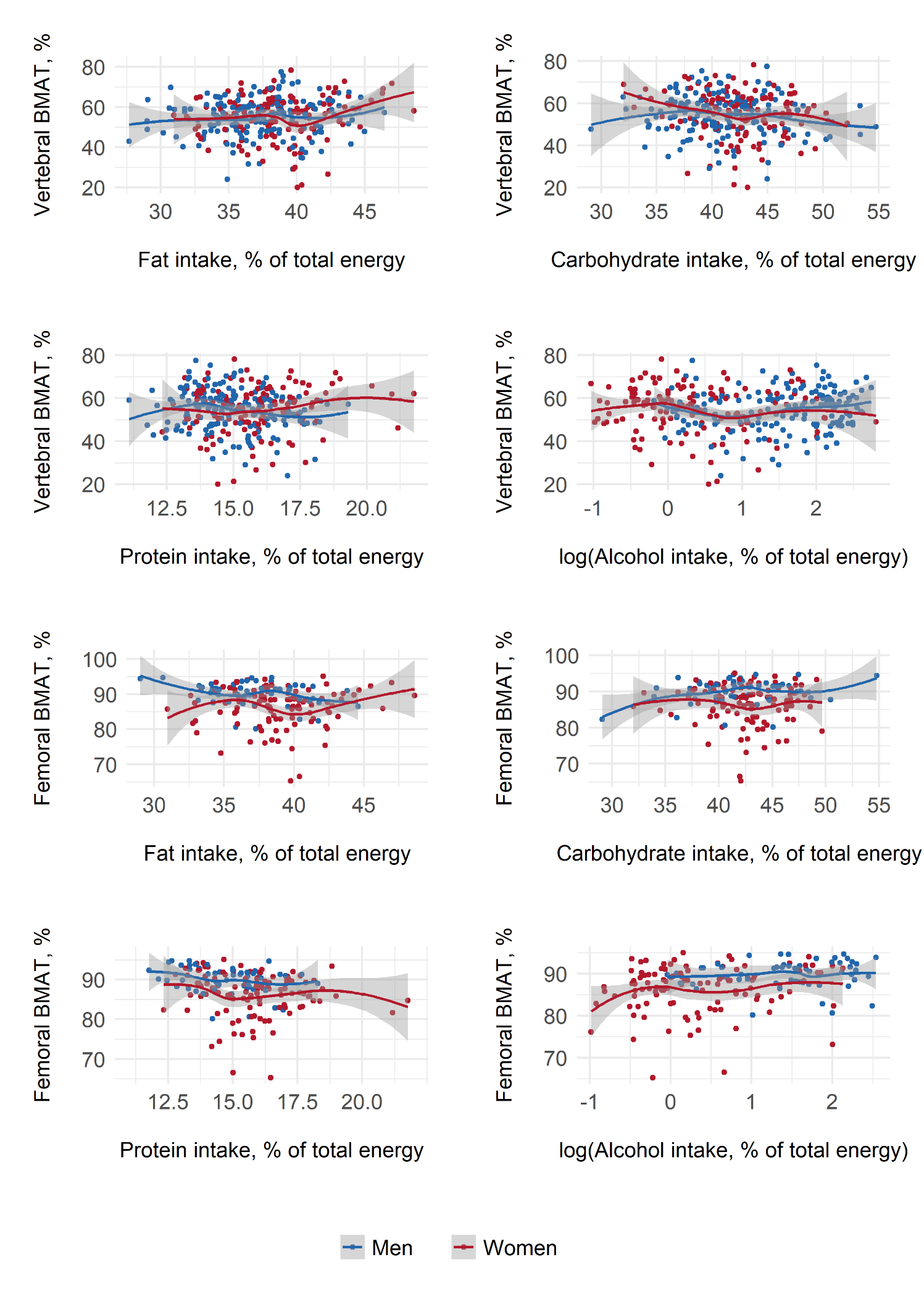


**Supplementary Figure 6: Correlation between micronutrients and femoral BMAT.** Sex-stratified scatterplots with linear smoothing.


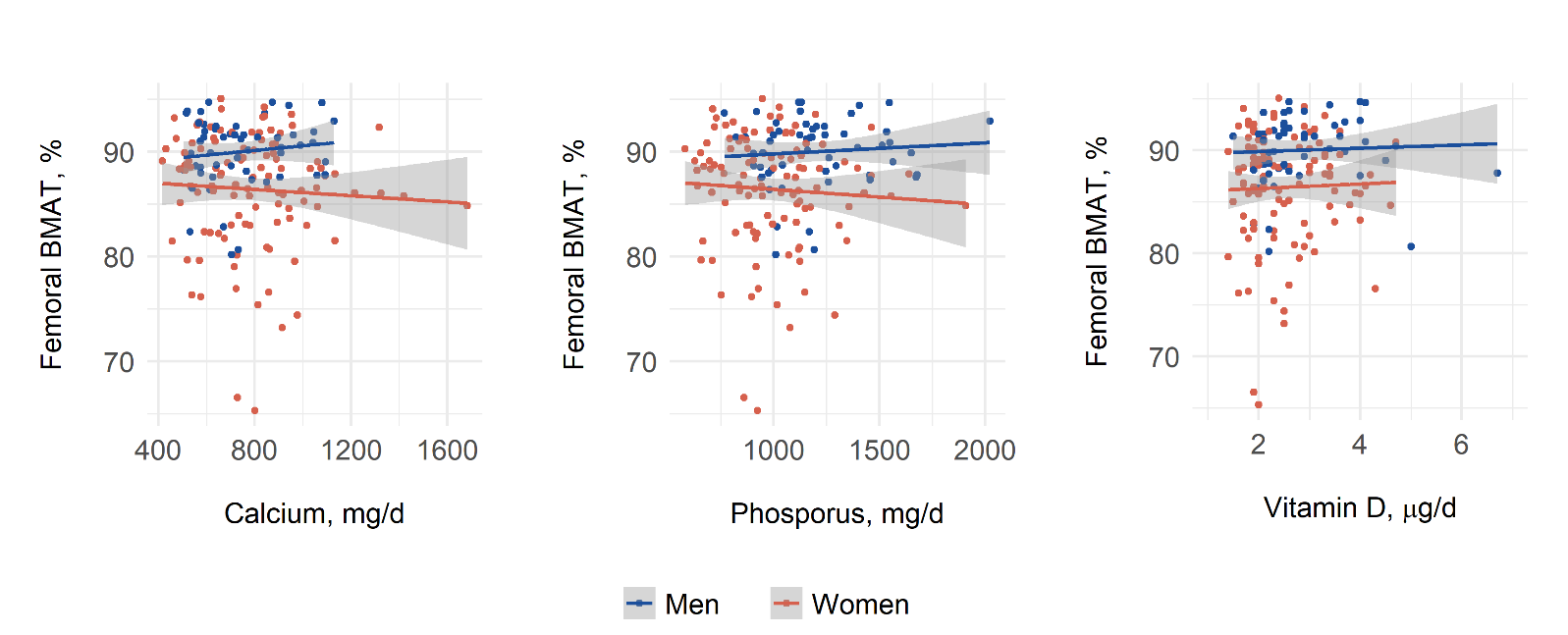

Supplement: Supplementary file 1 — Supplementary Material 1. [file 12937_2026_1365_MOESM1_ESM.docx]
